# Nodding syndrome in Uganda is a tauopathy

**DOI:** 10.1007/s00401-018-1909-9

**Published:** 2018-09-15

**Authors:** Michael S. Pollanen, Sylvester Onzivua, Janice Robertson, Paul M. McKeever, Francis Olawa, David L. Kitara, Amanda Fong

**Affiliations:** 1 Department of Pathobiology and Laboratory Medicine, University of Toronto, Toronto, ON Canada; 2 Ontario Forensic Pathology Service, Toronto, ON Canada; 3 Department of Pathology, College of Health Sciences, Makerere University, Kampala, Uganda; 4 Tanz Centre for Research in Neurodegenerative Diseases, University of Toronto, Toronto, ON Canada; 5 Department of Pathology, Gulu University, Gulu, Uganda; 6 Department of Surgery, Gulu University, Gulu, Uganda

**Keywords:** Neurodegeneration, Neurofibrillary tangles, Progressive supranuclear palsy

## Abstract

Nodding syndrome is an epidemic neurologic disorder of unknown cause that affects children in the subsistence-farming communities of East Africa. We report the neuropathologic findings in five fatal cases (13–18 years of age at death) of nodding syndrome from the Acholi people in northern Uganda. Neuropathologic examination revealed tau-immunoreactive neuronal neurofibrillary tangles, pre-tangles, neuropil threads, and dot-like lesions involving the cerebral cortex, subcortical nuclei and brainstem. There was preferential involvement of the frontal and temporal lobes in a patchy distribution, mostly involving the crests of gyri and the superficial cortical lamina. The mesencephalopontine tegmental nuclei, substantia nigra, and locus coeruleus revealed globose neurofibrillary tangles and threads. We conclude that nodding syndrome is a tauopathy and may represent a newly recognized neurodegenerative disease.

## Introduction

Nodding syndrome (NS) is a neurologic disorder of children in East Africa. NS is characterized by stereotypical head dropping movements, cognitive impairment, impaired growth, and seizures [2, 4, 5, 7, 16, 19, 23, 24]. The age of onset is usually 5–15 years of age [5]. Although NS has been documented in the Republic of South Sudan [2, 20, 23, 24] and Tanzania [19], it is currently a major health problem in the subsistence-farming villages of the Acholi people in northern Uganda [5, 7, 16]. NS emerged as an epidemic in the Kitgum district of Uganda in 1998 [5, 11] during internal armed conflict and displacement of children into camps. Over 3000 NS-affected children have been documented in northern Uganda [11]. Clinical descriptions of NS have focused on the seizures that are found in the disorder [2, 4, 7, 16, 19]. However, relentless neurologic deterioration [19] and death are often reported.

The cause of NS is not known. Different causal theories have been proposed including: onchocerciasis [4, 5, 20, 24], neurotropic virus [18], war-induced post-traumatic stress disorder [15], and environmental and nutritional factors [18, 20]. The current leading and controversial theory is that NS may be due to an autoimmune reaction to leiomodin-1 on the basis of a shared epitope in *Onchocerca volvulus* and the human brain [8].

No clinicopathologic studies have been published on NS. The aim of the current study was to investigate the neuropathologic basis of NS. On this basis, we now report the neuropathologic and immunohistochemical hallmarks of NS in five cases. We present histologic evidence the NS is a novel tauopathy.

## Methods

### Subjects

Five cases of NS who died in their villages in northern Uganda, or in a local hospital, in 2014 and 2017 were used for this study. All cases fulfilled the consensus-based diagnostic criteria for probable or confirmed NS [4]. Clinical history was obtained at the time of autopsy, or retrospective interview of the family (Table 1). Permission to conduct autopsies and publish the results was obtained from the affected families.

**Table 1:** Cases of nodding syndrome in this study

Characteristics	Case 1	Case 2	Case 3	Case 4	Case 5
Age at death (years)/sex	14/F	14/M	17/M	18/M	13/F
Year of death	2014	2014	2014	2017	2014
Age (years) at symptom onset	10	8	10	6	8
Year of symptom onset	2010	2008	2007	2005	2009
Duration of illness (years)	4	6	7	12	5
Location	Kitgum	Gulu	Kitgum	Kitgum	Pader
Place of symptom onset	IDP camp^a^	IDP camp	IDP camp	IDP camp	IDP camp
Antiepiletic drug therapy	Yes	Yes	Yes	Yes	Yes
General autopsy findings	Unkempt, malnourished, and dehydrated with multiple healing injuries	Multiple scars	Unkempt and malnourished	Unkempt and malnourished	Malnourished with multiple healing injuries
Immediate cause of death	Dehydration and malnutrition	Aspiration of gastric contents	Malnutrition	Lung abscess and empyema	Malnutrition

^a^Internally displaced person camp

### Histologic preparation

Histologic sections were obtained from formalin-fixed brains. In some cases, the brains had already been sectioned and blocks had already been sampled. The cerebrum was cut in the coronal plane, the brainstem sectioned in the horizontal plane, and the cerebellum sectioned sagittally and para-sagittally. Standardized blocks for paraffin embedding were obtained, if available, from the following areas: frontopolar cortex (Brodmann area 10); cingulate gyrus at the level of the head of the caudate nucleus; superior and middle frontal gyri at the level of the anterior striatum; superior and middle temporal gyrus at the lateral geniculate body; amygdala at the level of the anterior commissure; hippocampus and hippocampal gyrus; anterior striatum; inferior parietal lobule; calcarine cortex (Brodmann area 17); thalamus including subthalamic nucleus; midbrain at the level of the red nucleus; pons at the level of the locus coeruleus; medulla at the level of the olive; caudal medulla at the level of the nucleus cuneatus and gracilis; and cerebellum including the dentate nucleus. Sections were prepared for routine staining with Luxol fast blue with hematoxylin and eosin. Selected sections were stained with the Bielschowsky method.

### Immunohistochemistry

All brain areas were immunohistochemically studied with an antibody to abnormally phosphorylated tau (AT8, Invitrogen), using the immunoperoxidase technique. Selected brains areas including the cerebral cortex, brainstem and cerebellum were also immunostained with antibodies to α-synuclein (LB509, Novex), α-amyloid (6F/3D, Dako), and phosphorylated TDP-43 (MABN14, Milliepore). Sections of cerebral cortex from cases 2 were immunostained with antibodies to 3-repeat tau (RD3, Milliepore) and 4-repeat tau (RD4, Milliepore). Brain tissue from a case of Alzheimer’s disease, Lewy body-type Parkinson’s disease and frontotemporal degeneration with TDP-43 positive-inclusions were used as positive controls for immunoreactivity for the appropriate antibody. The primary antibody was omitted from the immunoperoxidase method for the negative control. The severity of AT8-immunoreactivity was graded on a four point scale: 0—none; +—mild; ++—moderate; and +++—marked.

## Results

### Clinical characteristics

**Fig. 1:**
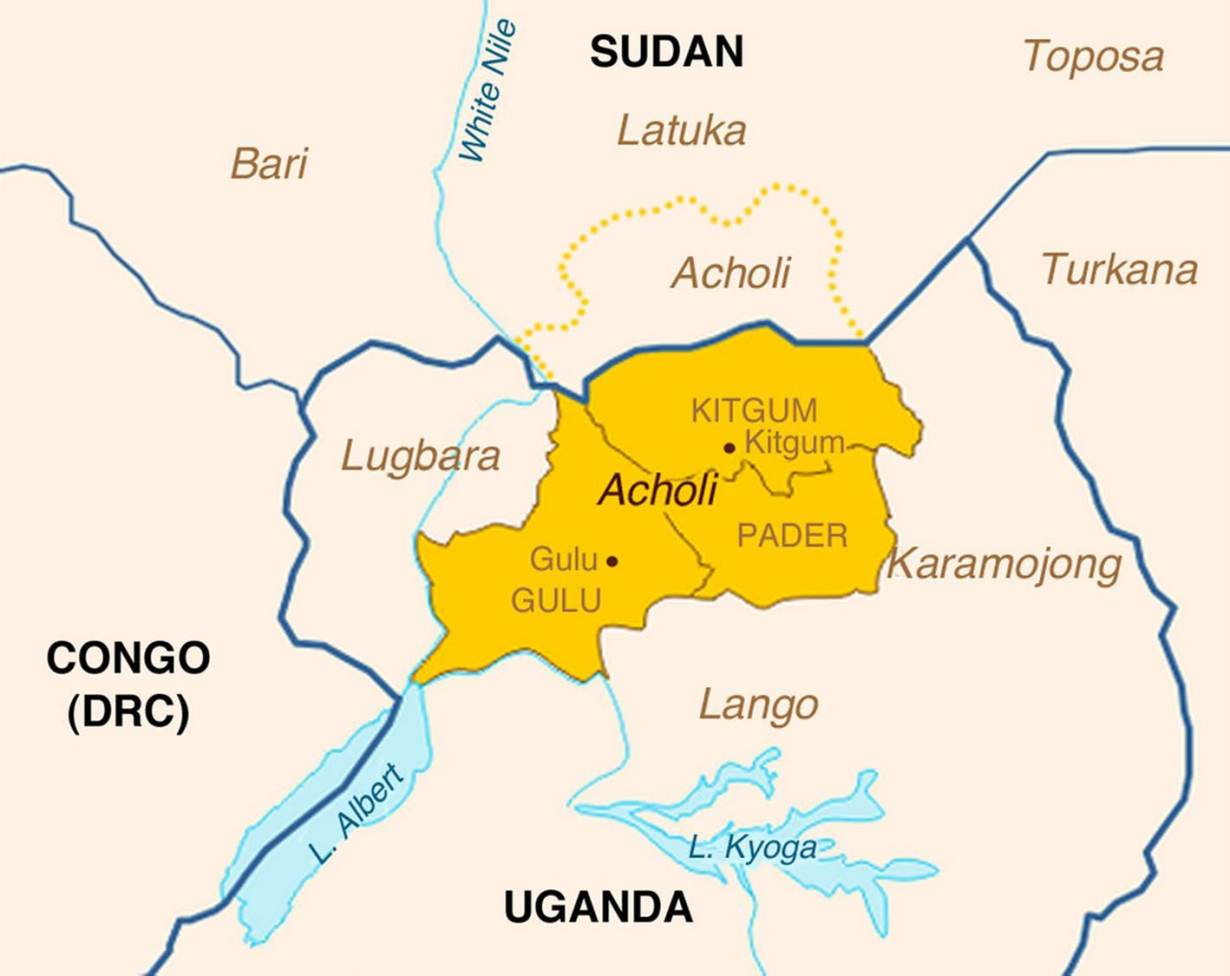
Three districts in northern Uganda affected by nodding syndrome [11]

All five cases derived from northern Uganda from the three districts inhabited by the Acholi people: Kitgum, Pader and Gulu (Fig. 1). The cases ranged from 13 to 18 years of age at death with three males and two females (Table 1). In all cases, the clinical symptoms were classical for NS with head dropping spells (nodding), cognitive impairment, seizures, and neurologic deterioration resulting in terminal wasting in four cases, and sudden death in one case. None of patients had chronic neurologic symptoms or signs before the onset of the symptoms of NS. All patients were treated with the anti-epileptic drugs carbamazepine and valproic acid. All patients developed symptoms following the height of the recent insurgency in northern Uganda and resided in camps for internally displaced people [11] at some time in the course of their illness. Three of the affected children had multiple injuries complicating seizure-related falls. Four of the children were malnourished and wasted due to the inability to swallow at the end stage of the illness. One child apparently died of aspiration of gastric contents secondary to a seizure (case 2), whereas the others died of a lung abscess or malnutrition.

### Neuropathologic findings

**Fig. 2:**
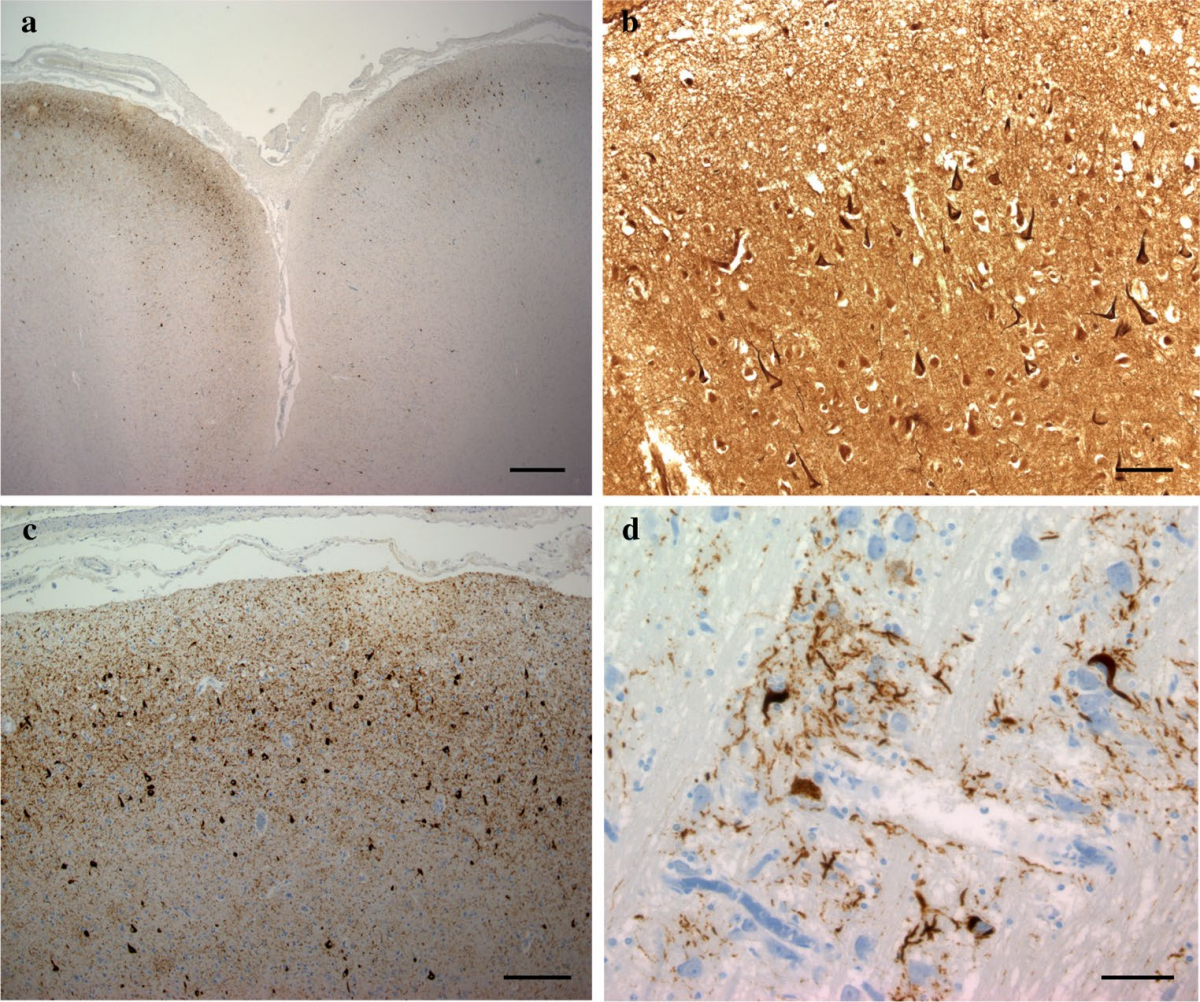
Histologic findings in nodding syndrome. **a** Tau-immunoreactive in frontal cortex, mostly in gryal crowns (AT8, scale bar: 1000 μm). **b** Cortical neurofibrillary tangles (Bielschowsky stain, scale bar: 100 μm). **c** Neurofibrillary tangles, dystrophic neurites and dot-like immunoreactivity containing phosphorylated tau in cerebral cortex (AT8, scale bar: 200 μm). **d** Neurofibrillary tangles and dystrophic neurites in neurons in the base of pons (AT8, scale bar: 75 μm)

All five cases showed the same pathologic findings in the same anatomical distribution with some variation in severity (Table 2). Macroscopically, there was mild frontotemporal cortical atrophy. On routine staining, the cerebral cortex showed superficial microvacuolation with neuronal neurofibrillary tangles, in all cases, with patchy frontal cortical status spongiosus (cases 2–5). The tangles ranged from argentophilic cytoplasmic wisps to classical flame-shaped tangles. Immunostaining for phosphorylated tau revealed tangles, pre-tangles (diffuse perikaryal and dendritic immunoreactivity), dot-like immunoreactivity in the neuropil, and neuropil threads (Fig. 2). The tangles, pre-tangles, dot-like lesions and threads were found mostly in the upper cortical lamina and tended to cluster. These changes were extensive but multifocally distributed in discontinuous bands, chiefly involving gyral crowns and less frequently in the depths of sulci. There was no perivascular predilection. Ghost tangles were often found in the superficial lamina. In areas of neocortex with the most tangles, all cortical lamina contained tangles with extensive involvement of pyramidal cells. Tangles, pre-tangles, dot-like lesions and threads were differentially distributed in the cortex. The most severe changes were present in the prefrontal cortex, and the superior and middle frontal gyri. The superior and middle temporal gyri and focally the parietal cortex were extensively involved. The occipital lobe was relatively spared. There was complete sparing of the hippocampus with minimal involvement of the cingulate gyrus, amygdala and hippocampal gyrus.

**Table 2:** Type, distribution and grade of histologic finding in cases of nodding syndrome

Site	Case 1		Case 2		Case 3		Case 4		Case 5	
	Neurofibrillary tangles and pre-tangles	Neuropil threads and dots	Neurofibrillary tangles and pre-tangles	Neuropil threads and dots	Neurofibrillary tangles and pre-tangles	Neuropil threads and dots	Neurofibrillary tangles and pre-tangles	Neuropil threads and dots	Neurofibrillary tangles and pre-tangles	Neuropil threads and dots
Frontal cortex	++	++	+++	+++	+++	+++	+++	+++	+++	+++
Temporal cortex	+	+	++	++	+++	+++	++	++	+++	+++
Parietal cortex	++	++	++	++	++	++	++	++	++	++
Occipital cortex	−	−	++	++	++	++	+	+	−	−
Motor cortex	−	−	+++	+++	NA	NA	NA	NA	++	++
Cingulate gyrus	+	+	+	+	++	++	+	+	+	+
Amygdala	−	−	+	+	+	+	+	+	+	+
Hippocampus	−	−	−	−	−	−	−	−	−	−
Hippocampal gyrus	+	+	+	+	+	+	+	+	+	+
Caudate nucleus	−	−	−	−	+	+	+	+	+	+
Putamen	−	−	−	−	+	+	−	+	+	+
Globus pallidus	−	−	−	−	++	++	−	−	−	−
Thalamus	+	+	−	−	++	++	+	+	+	+
Hypothalamus	+	+	+	+	NA	NA	NA	NA	+	+
Substantia nigra	+	+	+	+	NA	NA	+	+	++	++
Red nucleus	NA	NA	+	+	NA	NA	−	−	+	+
Colliculi	NA	NA	−	−	NA	NA	−	−	+	+
Periaqueductal grey matter	NA	NA	−	−	NA	NA	−	−	+	+
Mesencephalopontine tegmental nuclei	+	+	+	+	NA	NA	++	++	++	++
Locus coeruleus	NA	NA	+	+	++	++	++	++	NA	NA
Pontine nuclei	−	−	++	++	++	++	++	++	NA	NA
Inferior olivary nucleus	−	−	−	−	−	+	NA	NA	NA	NA
Medullary tegmental nuclei	−	−	+	+	+	+	NA	NA	−	++
Cerebellum	−	−	−	−	−	−	−	−	−	−
Dentate nucleus	−	−	−	−	−	−	−	−	+	+

*NA* not available

Globose tangles, pre-tangles and threads were found in various subcortical and brainstem sites (Fig. 2). The tegmental nuclei of the upper brainstem, including the Edinger–Westphal nucleus, substantia nigra, locus coeruleus and the nuclei of the pontine base were preferentially affected. Less extensive formation of tangles, pre-tangles and threads was found in the striatum, lentiform nuclei thalamus and medulla. There was relatively minimal or no involvement of the olives or dentate nuclei. Patchy to extensive Purkinje cell loss with Bergman gliosis and empty baskets in the cerebellum were found in three cases (cases 2, 3 and 5). There was moderate diffuse pallor of myelin staining in one case with preferential involvement of subcortical white matter and the pontocerebellar fibres (case 2). In severely involved areas of neocortex, there were tau-immunoreactive threads in subcortical white matter. Glial cellular inclusions were not prominent in any brain; however, occasional fuzzy/granular astrocytes were found in the locus coeruleus in case 4.

The tangles were immunoreactive with antibodies to 3-repeat and 4-repeat tau. No immunostaining was found with antibodies to α-synuclein, TDP-43, or β-amyloid. Other pertinent negative observations include no ballooned neurons, no Pick bodies, no Lewy bodies, no neuritic plaques, and no spongiform change. The pathologic features of subacute sclerosing panencephalitis, or other viral or autoimmune encephalitis were not present.

## Discussion

The hallmarks of NS include widespread formation of tangles, pre-tangles, neuropil threads and dot-like tau deposits in the cerebral cortex and brainstem with lesser involvement of the basal ganglia. The cortical involvement shows a frontotemporal predilection but parietal lobe involvement is also present. The prefrontal cortex and the anterior frontal lobe are the most severely affected. The brainstem involvement is principally centred on the substantia nigra, locus coeruleus, tegmental nuclei and the nuclei of the pontine base. These findings indicate that NS is a tauopathy. Based on the progressive clinical course and the nature of the histologic findings, NS may be a newly recognized neurodegenerative disease. Cerebellar degeneration was also present in three of the five cases, and could be explained by four different mechanisms: the direct effect of the NS, hypoxia secondary to seizures, anti-epileptic medication, or a combination of mechanisms. However, the cerebellar degeneration is likely related to the anti-epileptic medication because the severity of the tau-burden did not correlate with presence of cerebellar degeneration, (e.g., case 4 had a substantial tau-burden, but lacked cerebellar degeneration) and cerebellar ataxia is not a documented clinical feature of NS.

It is interesting to compare and contrast NS with other tauopathies. The distribution and cellular pathology in NS does not precisely match any other known tauopathy. The patchy cortical involvement in NS is similar to chronic traumatic encephalopathy [13]. However, the distribution of pathology within the cortex is different. In NS, the crests of gyri, rather than the depths of sulci, are preferentially involved and there is no perivascular predilection [12]. In addition, there is some overlap between the cortical deposition of tau in NS and temporal lobe epilepsy [21]. However, numerous cortical and subcortical tangles are not reported to occur in epilepsy, but these are distinctive findings in NS. Furthermore, a detailed mapping of tangles and tau-immunoreactivity, beyond the temporal lobe, in cases of epilepsy has found a propensity for a limbic distribution [22]. There is dissimilarity in the distribution of pathology of our five cases of NS. Thus, it is unclear if repeated seizures can explain the widespread tauopathy in NS. One possibility is that the cortical tauopathy form epileptogenic foci that cause the ongoing seizures in NS. However, if repeated seizures can cause widespread cortical and subcortical tangles and other tau-immunoreactive lesions, then this could be an alternate explanation.

NS also shares some pathological features with progressive supranuclear palsy due to a similar distribution of tangles [3]. However, the glial pathology in this series of NS cases is limited and was represented by granular/fuzzy astrocytes in the locus coeruleus in case 4. In addition, the tau deposits had both 3-repeat and 4-repeat tau isoforms, rather than selective enrichment of 4-repeat tau that occurs in progressive supranuclear palsy [1]. Case 4 was the oldest child in the series and had the longest duration of disease. The presence of granular/fuzzy astrocytes is similar to that described in ageing-related tau astrogliopathy [10]. Further characterization of the potential spectrum of astrogliopathy in a larger number of NS cases, at different stages of disease, is indicated. In particular, it will be interesting to determine if granular/fuzzy astrocytes develop into tufted astrocytes [9].

The clinical descriptions of NS have described cognitive impairment [16] and Parkinsonism [19]. But, no studies have described the late stages of disease progression in NS. Although the tegmentum, locus coeruleus and substantia nigra contain tangles and threads, neuronal loss and gliosis were not prominent features in five cases reported herein. Further histologic studies of the brainstem of in a larger cases series of NS with clinicopathologic correlation will be informative. Specifically, neurologic assessment for supranuclear palsy and extra-pyramidal signs in late-stage cases of NS with correlation with brainstem lesions will be important.

There are both similarities and differences between NS and the classical disease described by Hirano on Guam [6], and that was also later recognized in other Western Pacific foci [17]. The main similarities are that both disorders emerged as epidemics in indigenous people at a particular time and place, and both diseases have abundant tangles. In addition, both diseases are fading epidemics of unknown cause. The main differences are that NS appears to be purely a tauopathy, whereas the Guam disease is a polyproteinopathy [14], and the age of onset and clinical presentations are different. In addition, the Guam disease has extensive limbic involvement [6], whereas NS does not.

The cause of NS is not apparent from our neuropathological findings. However, there was no histologic evidence of viral or autoimmune encephalitis, or cerebral parasitism. The overall pathological process was neurodegeneration with tau-deposition. Therefore, if NS is caused by infection with a virus, or other infectious agent, then the nexus is unlikely through the pathogenic mechanisms that cause a post-encephalitic state, as occurs in measles infections leading to subacute sclerosing panencephalitis.

In conclusion, we have shown that the epidemic neglected tropical disease known as NS is a tauopathy. On this basis, NS may be a newly recognized neurodegenerative disease with a regional cluster in East Africa. The discovery that NS is a tauopathy may facilitate determining the cause of NS, because it opens up new lines of inquiry that were unavailable prior to our neuropathologic studies.
